# Feasibility of repairing skin defects by VEGF_165_ gene‐modified iPS‐HFSCs seeded on a 3D printed scaffold containing astragalus polysaccharide

**DOI:** 10.1111/jcmm.17800

**Published:** 2023-06-01

**Authors:** Weibin Du, Jintao Hu, Xiaolong Huang, Zhenwei Wang, Huateng Zhou, Yadong Yang, Huahui Hu, Rongliang Chen, Fuxiang Shen, Renfu Quan

**Affiliations:** ^1^ Research Institute of Orthopedics The Affiliated Jiangnan Hospital of Zhejiang Chinese Medical University Hangzhou China; ^2^ Hangzhou Xiaoshan Hospital of Traditional Chinese Medicine Hangzhou China; ^3^ Orthopedics and Traumatology Department Hangzhou Traditional Chinese Medicine Hospital Affiliated to Zhejiang Chinese Medical University Hangzhou China; ^4^ School of Laboratory Medicine and Bioengineering Hangzhou Medical College Hangzhou China

**Keywords:** 3D printed degradable scaffold, astragalus polysaccharide, hair follicle stem cells, induced pluripotent stem cells, regeneration and repair, skin defect

## Abstract

The preparation of biodegradable scaffolds loaded with cells and cytokine is a feature of tissue‐engineered skin. IPSCs‐based tissue‐engineered skin treatment for wound repair is worth exploring. Healthy human skin fibroblasts were collected and reprogrammed into iPSCs. After gene modification and induction, CK19^+^/Integrinβ1^+^/CD200^+^ VEGF_165_ gene‐modified iPS‐HFSCs^GFP^ were obtained and identified by a combination of immunofluorescence and RT‐qPCR. Astragalus polysaccharide‐containing 3D printed degradable scaffolds were prepared and co‐cultured with VEGF_165_ gene‐modified iPS‐HFSCs^GFP^, and the biocompatibility and spatial structure of the tissue‐engineered skin was analysed by cell counting kit‐8 (CCK8) assay and scanning electron microscopy. Finally, the tissue‐engineered skin was transplanted onto the dorsal trauma of nude mice, and the effect of tissue‐engineered skin on the regenerative repair of total skin defects was evaluated by a combination of histology, immunohistochemistry, immunofluorescence, RT‐qPCR, and in vivo three‐dimensional reconstruction under two‐photon microscopy. CK19^+^/Integrinβ1^+^/CD200^+^ VEGF_165_ gene‐modified iPS‐HFSCs^GFP^, close to the morphology and phenotype of human‐derived hair follicle stem cells, were obtained. The surface of the prepared 3D printed degradable scaffold containing 200 μg/mL astragalus polysaccharide was enriched with honeycomb‐like meshwork, which was more conducive to the proliferation of the resulting cells. After tissue‐engineered skin transplantation, combined assays showed that it promoted early vascularization, collagen and hair follicle regeneration and accelerated wound repair. VEGF_165_ gene‐modified iPS‐HFSCs^GFP^ compounded with 3D printed degradable scaffolds containing 200 μg/mL astragalus polysaccharide can directly and indirectly participate in vascular, collagen, and hair follicle regeneration in the skin, achieving more complete structural and functional skin regenerative repair.

## 1 INTRODUCTION

The skin, as the first protective barrier between the outside world and the body, is an important part of the immune system.^1^, ^2^ The skin consists of the epidermis, dermis, and subcutaneous tissues, and has defence, immunity, regulation of body temperature, and metabolism.^4^, ^5^, ^6^ However, skin trauma or soft tissue injury from various clinical causes, such as trauma, burns, contusions, etc., is becoming common. With the increasing global trend of aging, the consequent problem of skin injury is gradually increasing, especially the trauma care and healing issues are thus receiving more attention. Medical dressings and skin substitutes have significant advantages in accelerating skin wound healing and preventing and treating skin infections. It is estimated that the trade of medical dressings have reached nearly 23.8 billion yuan in 2021.^7^, ^8^, ^9^

Tissue‐engineered skin, as a skin substitute, is a biodegradable scaffolds prepared by engineering means, which is also loaded with cells and cytokine, etc. The scaffolds degrade themselves during the process of cell growth and differentiation and tissue formation to form an autologous skin. This provides a new method for constructing skin substitutes with good development prospects and also has very important clinical significance in producing more desirable therapeutic effects for people with difficult wound healing. First, screening for suitable seed cells is considered one of the simplest and most effective methods in tissue‐engineered skin construction,^10^, ^11^, ^12^ and hair follicle stem cells (HFSCs) are present in the ramus of the outer root sheath of the hair follicle, and their specific markers include cytokeratin 19 (CK19),^13^, ^14^ cell membrane protein CD200,^15^, ^16^ and integrin β1 (ITGβ1).^17^, ^18^ HFSCs have the ability of self‐renewal and proliferation in vitro and have the potential to differentiate into hair follicle tissue, connective tissue, neuronal cells, epidermal layer cells and melanocytes, and vascular smooth muscle cells.^19^, ^20^, ^21^ VEGF_165_ is one of the five subtypes of VEGF, with the strongest activity and the widest distribution. It plays an important role in angiogenesis, formation and induction of endothelial cell differentiation. Our research group has successfully constructed a hair follicle stem cell system with VEGF_165_ overexpression in previous experiments. However, there are some problems such as insufficient efficiency of hair follicle stem cell acquisition, easy cell senescence, and more difficult control of transgenerational digestion,^22^, ^23^ which greatly limit their clinical applications, so obtaining enough and pure hair follicle stem cells for constructing tissue‐engineered skin remains a challenge.

IPSCs have theoretically unlimited proliferative capacity and can differentiate into nearly all adult cell types, and are considered to be seed cells for the treatment of many diseases. Skin fibroblast is relatively easy to obtain and is a adult seed cell reprogrammed into iPSCs earlier. The reprogramming technique, introducing specific transcription factors OCT4, SOX2, C‐MYC, and KLF4 into adult seed cells to transform it into iPSCs,^24^, ^25^ overcomes the ethical hurdles of traditional pluripotent stem cell research and has the advantages of being widely available, easy to obtain, and the ability to obtain normal or disease models, and is useful in regenerative medicine for with great therapeutic promise. The successful differentiation of iPSCs into hair follicle stem cells has been reported in the literature, mainly through suspension and adherent culture systems. Bone morphogenetic protein‐4 (BMP‐4), retinoic acid (RA) and epidermal growth factor (EGF) are key cytokine for iPSCs differentiate into skin epithelium. BMP‐4 can induce epithelial differentiation and block neural differentiation. When BMP‐4 blocks neural differentiation, RA induces cell differentiation in epithelial cells. RA and EGF in turn differentiate iPSCs into ectodermal and mesodermal cells.^26^, ^27^, ^28^ In our group, based on the reported protocols in the literature and the use of the above‐mentioned cytokines in the early stage, although iPSCs induced CD200^+^/ITGA6^+^ epithelial stem cells exhibited a hair follicle stem cell phenotype, the positive rate was still only about 20% and the induction protocol was relatively cumbersome.

The use of 3D printed technology and herbal monomers in skin wound repair has become a new hotspot. The skin bioactive scaffold can provide a suitable three‐dimensional space for seed cells to grow, thus facilitating a good bioactive environment for skin wound healing.^29^, ^30^ Compared with traditional preparation techniques such as the freeze‐drying method, electrostatic spinning method, and pore‐making agent method, 3D printed bioactive scaffolds can be more efficiently prepared to meet the requirements of skin wound repair. 3D printed technology is highly flexible and reproducible and can be directly preset to meet the skin cell growth porosity for batch 3D structure printing. Astragalus polysaccharide has been found to have a strong pro‐vascular regenerative effect, which accelerates trauma repair.^31^, ^32^ Collagen has low immunogenicity and can provide a microenvironment for cell apposition, growth, proliferation and directed differentiation.^33^, ^34^ Sodium alginate is water‐soluble alginate that facilitates the maintenance of a moist wound environment and absorbs wound exudate, which also increases the formability of the scaffold.^35^, ^36^ Silk fibroin is a natural protein with good biocompatibility and mechanical properties.^37^

Therefore, we constructed a normal human skin fibroblast‐derived iPSCs system. The iPSC‐derived VEGF_165_ gene‐modified iPS‐HFSCs^GFP^ system was constructed using a modified induction method. Astragalus polysaccharide‐collagen‐sodium alginate‐silk fibroin 3D printed degradable skin scaffolds were prepared and constructed with VEGF_165_ gene‐modified iPS‐HFSCs^GFP^ co‐culture system. This tissue‐engineered skin was transplanted into the nude mice with dorsal whole skin defect wounds to explore and analyse its role effectively, form of action and mechanism of action in accelerating wound repair.

## 2 METHODS

### 2.1 Experimental animals

Thirty‐six SPF‐grade BALB/c nude mice, both male and female, weighing 10–20 g, were housed at the Animal Experimental Research Center of Zhejiang University of Traditional Chinese Medicine, with experimental animal use permit No. SYXK (Zhejiang) 2021‐0012. purchase and feeding, and other animal procedures followed the animal research guidelines of the National Institute of Health and the Animal Research Committee. It was approved by the Experimental Animal Ethics Committee of the Zhejiang University of Traditional Chinese Medicine (No. IACUC‐20210927‐07).

### 2.2 Reagents

The main reagents include DMEM/F12 (1:1) medium, knockout serum replacement (KSR), valproic acid, fetal bovine serum, Essential 8™ Flex Medium, Geltrex™, KSFM medium, BMP‐4, bovine pituitary extract (BPE), (Gibco), NANOG, OCT4, SOX2, SSEA‐4, TRA‐1‐81, TRA‐1‐60, Krt19, Integrinβ1, CD200, CD31 and VEGF‐A antibodies (Abcam), PDGF‐B and Ang2 antibodies (Santa Cruz), recombinant human EGF (R&D), RA, valproic acid, sodium alginate (Sigma), Matrigel^®^ Matrix (Corning), mTeSRTM1 medium (Stem Cell), Astragalus polysaccharide (Solarbio), silk fibroin and collagen (Hefei Bomei Bio), and Dextran Texas red™ (Invitrogen). Primers were designed using Primier 6.0 software, and all primers were synthesized as shown in Table 1.

**TABLE 1:** Primer sequences for reverse transcription‐polymerase chain reaction.

Gene size (bp)	5′–3′	Primers sequences	Fragment
Nanog	Forward	ACCTATGCCTGTGATTTG	170
	Reverse	AGAAGTGGGTTGTTTGC	
CK19	Forward	ACAGCCACTACTACACGACCA	169
	Reverse	ATGTCGGCCTCCACGCTCA	
Integrinβ1	Forward	TGTTCTTATTGGCCTTGCAT	168
	Reverse	TCATACTTCGGATTGACCAC	
CD200	Forward	CCCAGCCCTATTTTACGTC	107
	Reverse	CATATGAATTGGCTGACTGCT	
VEGF_165_	Forward	AAAGTCTAGCCCTGTTTCGG	108
	Reverse	CCTCAAATTAGCCAATGGTG	
CD31	Forward	AGCCAGCAGTATGAGGACCAGTC	100
	Reverse	TCCAATGACAACCACCGCAATGAG	
PDGF B	Forward	TCTCTGCTGCTACCTGCGTCTG	145
	Reverse	AGCCCCATCTTCATCTACGGAGTC	
VEGF A	Forward	GGGCTCTTCTCGCTCCGTAGTAG	137
	Reverse	CCCTCTCCTCTTCCTTCTCTTCCTC	
Ang‐2	Forward	TCCAACACCGAGAAGATG	210
	Reverse	CACCAGCGAGGTAGAAGT	
a‐SMA	Forward	CGTGGCTATTCCTTCGTGACTACTG	148
	Reverse	CGTCAGGCAGTTCGTAGCTCTTC	
Actin	Forward	GCTATGTTGCCCTAGACTTCGA	173
	Reverse	GATGCCACAGGATTCCATACC	

### 2.3 Generation and identification of IPSC

The frozen human skin fibroblasts from our group were revived and induction of iPSCs refers to early mature methods of our group.^38^, ^39^ Briefly, four plasmids (PCXLE‐hOCT3/4‐shp53‐F, PCXLE‐hSK, pCXLE‐hUL, and pCXWB‐EBNA1) were transfected into human skin fibroblasts by electrotransfection, and the electrotransferred cells were cultured for about 7 days. The cells were then cultured into a culture dish precoated with mouse embryo fibroblast (MEF) dishes until iPS clone clusters were formed. Individual clone clusters are selected and transferred to a new MEF feeder layer or cultured in matrigel‐coated dishes for expansion and can continue to be cultured in the finished mTeSR1 medium of choice. Collect iPSCs in good condition and freeze them in liquid nitrogen for storage. Under the microscope, monoclonal cells in good condition were selected and subjected to alkaline phosphatase activity (ALP) staining assay.

The cells were collected and six specific proteins Nanog, OCT4, SOX2, SSEA‐4, TRA‐1‐81, and TRA‐1‐60 were detected by immunofluorescence. Cells were plated onto slides at a concentration of 1.0 × 10^5^ cell/well for cell crawling, fixed in 4% paraformaldehyde‐PBS for 10 min at room temperature, 0.25% TritonX100‐PBS for 10 min at room temperature for cell permeabilization experiments, and 5% bovine serum albumin BSA‐PBS for 30 min at room temperature for cell closure. primary antibody was added at a concentration of 1:100. Then incubated at 4°C overnight. Fluorescein‐labelled secondary antibody was added and incubated for 30 min at room temperature, protected from light. nuclei were stained with DAPI (1:2000), blocked and photographed.

IPSCs were cultured in suspension and morphological changes of embryoid bodies differentiation were observed. Matrigel gel mixture containing iPSCs was injected subcutaneously into the back of NOD‐SCID immunodeficient mice for in vivo teratoma formation assay. 10 weeks later, tumours were isolated for haematoxylin–eosin (H&E) staining to observe the presence of three germ layers of tissue.

### 2.4 Generation and identification of VEGF_165_ gene modified iPS‐HFSCs^GFP^

VEGF_165_ overexpressing lentiviral plasmid with green fluorescent protein (GFP):psPAX2:PMD2G = 4:3:1 was transfected into HEK293T cells to generate lentivirus. GFP fluorescence intensity was observed under a fluorescence microscope, and transfection efficiency was calculated by taking into account the GFP fluorescence. GFP fluorescence intensity was observed under a fluorescence microscope, and transfection efficiency was calculated by randomly taking eight 200× microscopic fields. RT‐qPCR and Western blot were performed to detect VEGF_165_ expression in iPSCs and evaluate the overexpression efficiency.

Referring to the induction procedure in the literature,^40^ cells were gently resuspended with 2 mL of Essential 8 medium containing RevitaCell™ supplement. Cells were plated onto pre‐Geltrex™ LDEV‐Free growth factor‐incubated plates and maintained in Essential 8 medium. After 2 days of culture, the culture medium was changed to HFSCs induction medium culture A (KSFM + 0.2 ng/mL EGF + 30 μg/mL BPE + 1% penicillin–streptomycin + 10 ng/mL BMP‐4 + 1 μM RA). The induction medium was changed every other day. After 7 days of induction, approximately 100% of the induced cells were left to fuse and passaged. Between 7 and 14 days of induction, 0.25 μM valproic acid was added to the previous HFSCs induction medium to improve induction, forming a new HFSCs induction complete medium B (KSFM + 0.2 ng/mL EGF + 30 μg/mL BPE + 1% penicillin–streptomycin + 10 ng/mL BMP‐4 + 1 μM RA + 0.25 μM valproic acid). 14 days later, using the proven system for the expansion and purification of HFSCs passaged by our group, the single suspension of induced cells obtained by digestion and centrifugation was inoculated into type IV collagen‐coated culture dishes, and the cells were purified and collected using the differential apposition method. The changes in cell morphology after induction were observed under an inverted microscope and photographed and recorded.

Immunofluorescence detection of HFSCs target proteins and RT‐qPCR detection of target genes was performed as described previously. Then, the induced hair follicle stem cells were compared with three target genes (CK19, Integrinβ1, CD200) of human hair follicle stem cells.

### 2.5 Preparation of 3D printed scaffolds containing astragalus polysaccharide and co‐culture with VEGF_165_ gene modified iPS‐HFSCs^GFP^

Cells were cultured in 96‐well plates at a concentration of 1 × 10^5^ cells/mL, 100 μL per well, and astragalus polysaccharide was diluted with culture medium to a concentration of 400, 200, 100, 50, 25, 12.5, 6.25 μg/mL, and 200 μL per well, and 6 replicates were set up in each group. After culture 72 h, cell proliferation was detected by the CCK‐8 method. 10 μL of CCK‐8 reagent was added to each well, mixed well and placed in a CO_2_ incubator, and the optical density (OD) value was detected at 450 nm wavelength by enzyme marker after 4 h. The optimum concentration of astragalus polysaccharide was selected for subsequent experiments.

Weigh 0.8 g of sodium alginate and 0.8 g of silk fibroin, dissolved in 8 mL PBS. The above formulated and sterilized hydrogel was filled into the print cartridge for 3D printing. Printing parameters: side length 10 mm, layer height 0.1 mm, printing needle 0.41 mm, printing pressure: 160 kPa, number of bars per layer: 8, 4 layers in total, 90° turning angle printing, the barrel was kept at 37°C. Immediately after printing, 5% calcium chloride was added to cure the material, and 150 μL 0.2% collagen (containing 200 μg/mL astragalus polysaccharide) was added dropwise to the scaffold. Put into 37°C incubator for 2 h to make collagen change from liquid to gel, saline or PBS solution soaked, and put aside in 4°C refrigerator.

When co‐culture, about 1 × 10^6^ cells/mL of VEGF_165_ gene modified iPS‐HFSCs^GFP^ were palted on each scaffold, and after cultured in a 5% CO_2_ incubator, the number of cell morphology and distribution were observed by photographing under an inverted microscope, and the fluorescence intensity of the cells were observed under a fluorescence microscope. 10 μL CCK‐8 reagent was added in 96‐well plates at 1, 2, 3, 4, 5, 6, 7 and 8 days, respectively, and 6 replicate wells were set at each time point, incubated in the incubator for 2 h, and the OD value was detected at 450 nm by enzyme marker, and after getting the average value, the cell growth curves were plotted at different time points.

The above empty scaffolds and tissue‐engineered skin were fixed in 2.5% glutaraldehyde solution at 4°C overnight. After fixation in 1% starvation acid solution, the samples were dehydrated with gradient concentrations (including five concentrations of 30%, 50%, 70%, 80%, 90%, and 100%) of ethanol solution, and then treated with a mixture of ethanol and isoamyl acetate, among other steps. After critical point drying and platinum spraying, the internal structure and cellular state of the biological scaffolds were observed under scanning electron microscopy.

### 2.6 VEGF_165_ gene‐modified iPS‐HFSCs^GFP^ composite containing astragalus polysaccharide 3D printed skin scaffold to repair whole skin defects in nude mice

Six‐week‐old BALB/c nude mice were anaesthetised, and the surgical area was disinfected. Then a 1.0 × 1.0 cm square full‐layer skin defect trauma model was prepared on the back of the nude mice. According to the different wound treatment methods, 12 BALB/c nude mice were randomly divided into 3 groups: blank control group A (covered with petroleum jelly), empty scaffold group B (3D printed skin scaffold containing astragalus polysaccharide) and tissue‐engineered skin group C (VEGF_165_ gene‐modified iPS‐HFSCs^GFP^ + 3D printed skin scaffold containing astragalus polysaccharide). All were wrapped with disposable sterile gauze and elastic bandages and anti‐infected with postoperative gentamicin. The trauma area was measured and calculated for each group at 7 and 14 days, and the percentage of trauma healing at the corresponding time points was counted to analyse the trauma healing.

After 14 days, square‐shaped healed wounds of the original size were taken for histological examination (H&E staining and Masson staining), microscopic observation and image acquisition for analysis. The tissue specimens were subjected to immunohistochemistry and immunofluorescence (α‐SMA and CD31), observed under a fluorescence microscope and the images were collected for analysis. We also performed RT‐qPCR on the tissue specimens to analyse the changes in five target genes (CD31, PDGF B, VEGF A, Ang‐2, and a‐SMA). The related gene expressions were compared by the $2^{-∆∆C_{t}}$ method. Finally, in vivo 3D reconstruction of traumatic vessels and VEGF_165_ gene‐modified iPS‐HFSCs^GFP^ were performed. Specifically, after successful anaesthesia of nude mice, Texas Red dextran solution at a concentration of 0.5 mg/mL was injected from the orbit and administered at a concentration of 0.01 mL/g. The nude mice were rested for 15 min, and after the red dye was successfully labelled through the blood circulation and distributed throughout the body. After fixation, live nude mice were imaged in vivo using an Olympus orthotopic two‐photon microscope, and the FV1000 driver software was used to acquire images of traumatic blood vessels (red) and VEGF_165_ gene‐modified iPS‐HFSCs^GFP^ (green), and the images were synthesized in three dimensions by Imaris software.

### 2.7 Statistical analysis

Each experiment was repeated at least three times. Results are shown as mean ± standard deviation (s), and the Wilcoxon test and *t*‐test were used for comparison between groups. Differences were considered statistically significant at *p* < 0.05.

## 3 RESULTS

### 3.1 Generation and identification of iPSC

**FIGURE 1:**
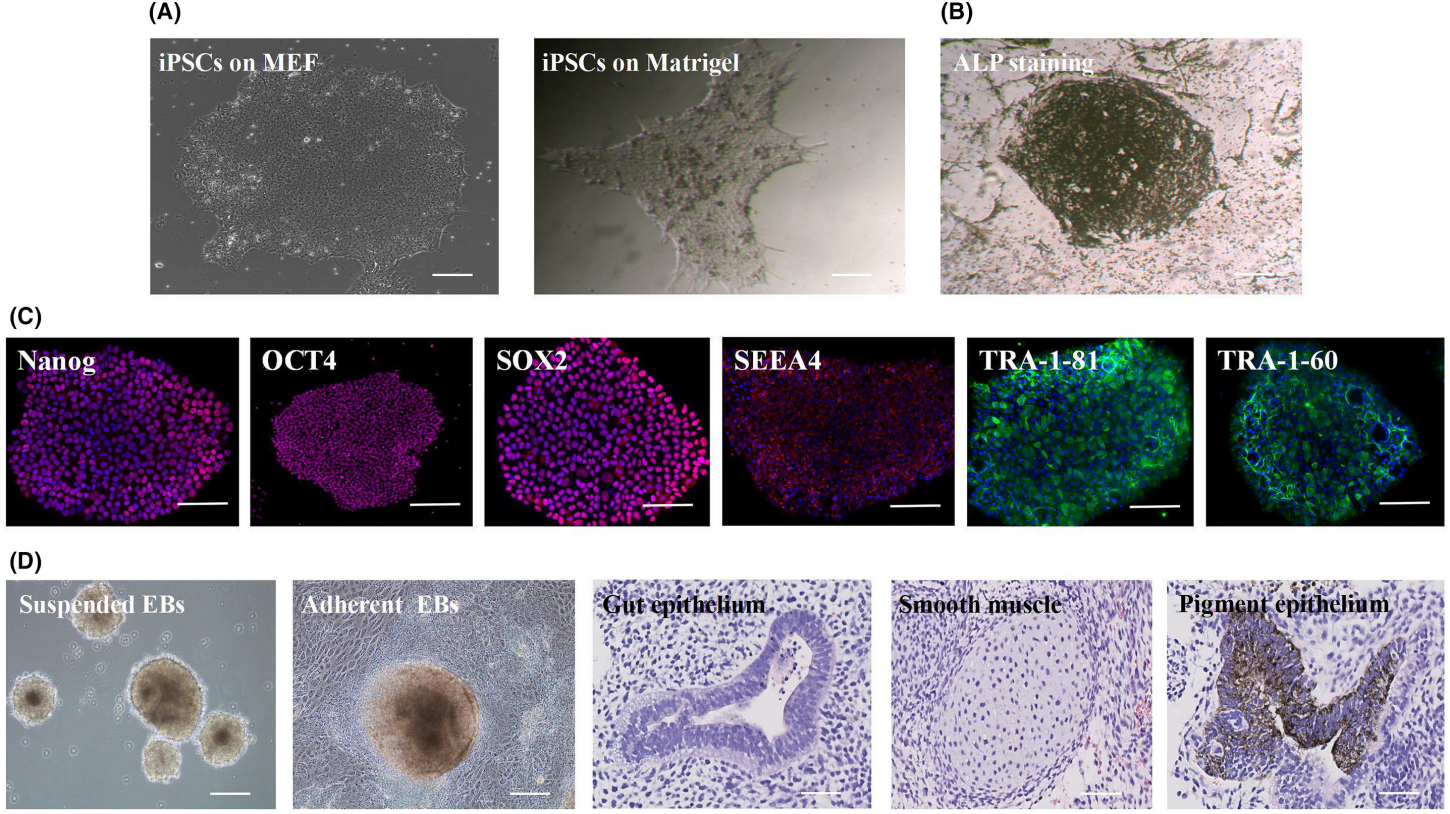
(A) Morphology of iPSCs on mouse embryo fibroblast feeder layers and in Martrigel gel‐coated Petri dishes. Scale bar, 100 μm. (B) Alkaline phosphatase staining of iPSCs. Scale bar, 100 μm. (C) Immunofluorescence staining of iPSCs‐specific markers. Scale bar, 200 μm. (D) Differentiation of iPSCs to mimic embryos. Scale bar, 100 μm. (E) H&E staining of teratoma showing the structure of the three germ layers. Scale bar, 100 μm.

Clone clusters appeared in the electrotransformed cells after 20 days. The clone clusters were palted onto feeder layer MEF and Matrigel gel, and microscopically, the cell morphology was similar to that of embryonic stem cells (ESCs), with clone clusters present in aggregates, in which the cells were closely arranged, with large nuclei, in good condition, with high clonogenic capacity and good viability (Figure 1A). iPSCs clone clusters with high ALP expression suggested that the cells were in the same undifferentiated state as embryonic stem cells, with high proliferative potential (Figure 1B). Immunofluorescence staining results showed positive expression of the allosteric specific proteins Nanog and OCT4 (red fluorescence). The specific markers cytosolic protein SOX2, and cell membrane protein SSEA‐4 were positively expressed (red fluorescence). This further confirmed that we have obtained human skin fibroblast‐derived iPSCs (Figure 1C). In vitro differentiation of embryoid bodies showed that the cells grew in suspension, in clusters, and gradually aggregated and grew larger on the second day. After 7 days, the embryoid bodies grew against the wall, and cells kept crawling out of the cell clusters with high proliferation capacity (Figure 1D). The results of teratoma formation identification experiments showed that the histological structures of the three embryonic layers could be observed under the microscope, especially the representative endoderm: intestinal epithelial cells; mesoderm: smooth muscle cells; and ectoderm: retinal pigment epithelial cells (Figure 1E). The above results demonstrated that we established a human skin fibroblast‐derived iPSCs system.

### 3.2 Generation and identification of VEGF_165_ gene modified iPS‐HFSCs^GFP^

#### 3.2.1 Results of the construction of VEGF_165_ overexpressed iPSCs^GFP^ line

**FIGURE 2:**
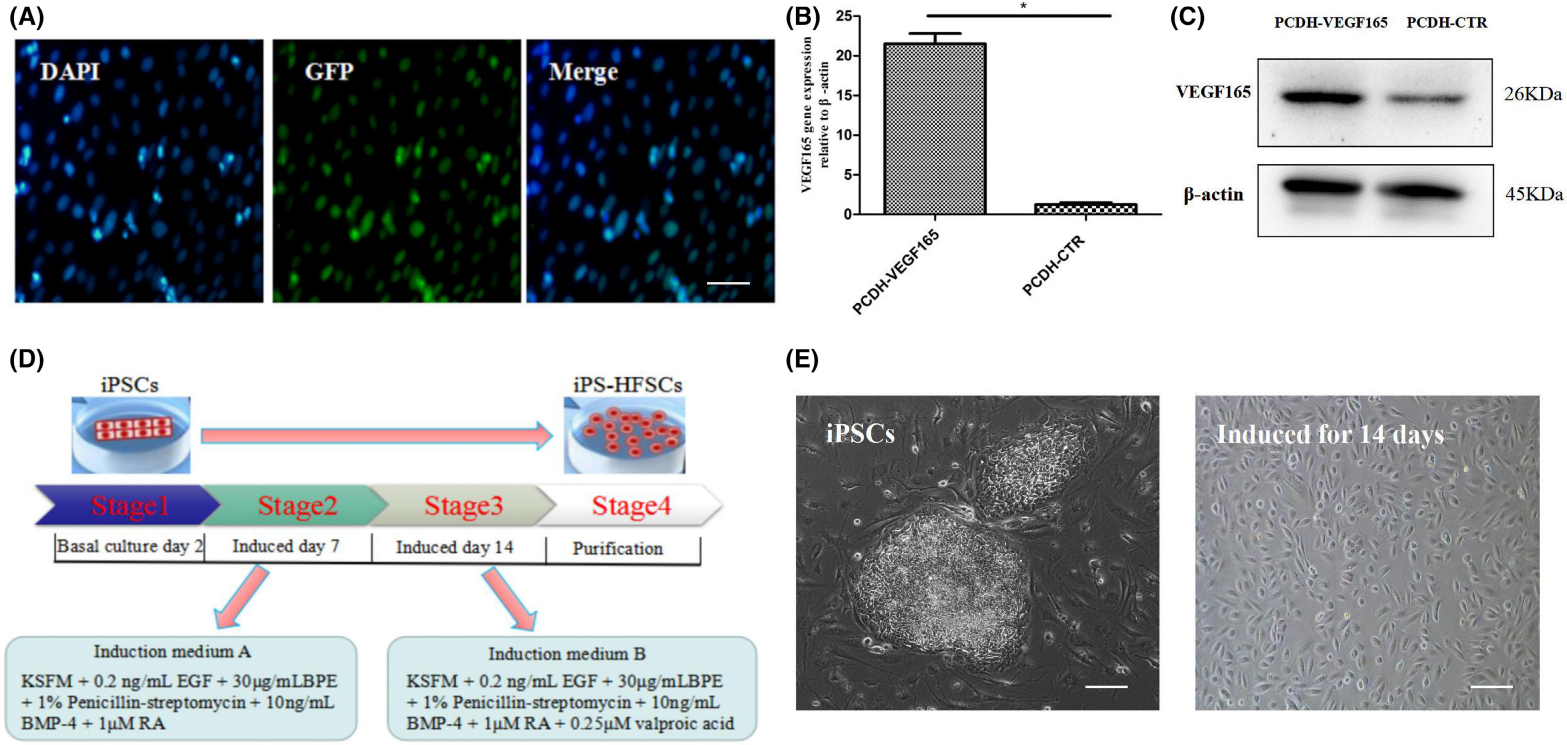
(A) Fluorescence microscopy results of GFP‐VEGF_165_ gene‐modified iPSCs. Scale bar, 100 μm. (B, C) Expression of VEGF_165_ gene and protein after iPSCs gene modification. (D) Flow chart of the differentiation system of GFP‐VEGF_165_ gene‐modified iPSCs to HFSCs. (E) Cell morphology at different differentiation stages. Scale bar, 100 μm.

Strong green fluorescence was observed in the experimental group (PCDH‐VEGF_165_) under a fluorescence microscope after 48 h. The lentiviral transfection efficiency reached 95.56% ± 1.64%, and the transfection efficiency was sustained and stable. The VEGF_165_ gene and protein expression of the experimental group (PCDH‐VEGF_165_) and the control group (PCDH‐CTR) were detected separately, and the results showed that the VEGF_165_ gene and protein expression of the experimental group (PCDH‐VEGF_165_) were significantly better than those of the control group (PCDH‐CTR), and the differences were statistically significant (*p* < 0.05; Figure 2A–C). The above indicates that the in vitro iPSCs^GFP^ line with VEGF_165_ overexpression was successfully constructed and VEGF_165_ was stably overexpressed.

#### 3.2.2 Induction process and morphological changes of generating VEGF_165_ gene modified iPS‐HFSCs^GFP^

According to the group's previous induction protocol and reference to the method of Benedetti et al.^27^ to induce iPSCs to differentiate into HFSCs, the specific protocol flow (Figure 2D): (1) VEGF_165_ overexpressed iPSCs were plated and cultured for 2 days. (2) KSFM was selected to induce differentiation toward epithelial cells and gradual formation of HFSCs by adding mainly 0.2 ng/mL EGF + 30 μg/mL BPE + 10 ng/mL BMP‐4 + 1 μM RA for 7 days. (3) Subsequently, iPSC‐derived HFSCs were maintained induced and expanded by adding 0.25 μM valproic acid for 7 days on top of this. (4) Purification of iPSC‐derived HFSCs.

Inverted microscopy showed that firstly iPSCs clone clusters were clearly present and the cells grew well. After 14 days of induction and expansion, the cells showed the typical pavement morphology of HFSCs with good stereoscopic sense and high refractive index, which had more typical biological properties of HFSCs (Figure 2E). Meanwhile, during the process of induction differentiation, we found that the cells' viability, proliferation power and overall cell status would decrease after 14 days. Therefore, we used the cells within 14 days of induction differentiation for cell phenotype detection and later construction of tissue‐engineered skin. The above results indicate that we have obtained cells with typical morphological characteristics of HFSCs.

#### 3.2.3 Immunofluorescence assay results

**FIGURE 3:**
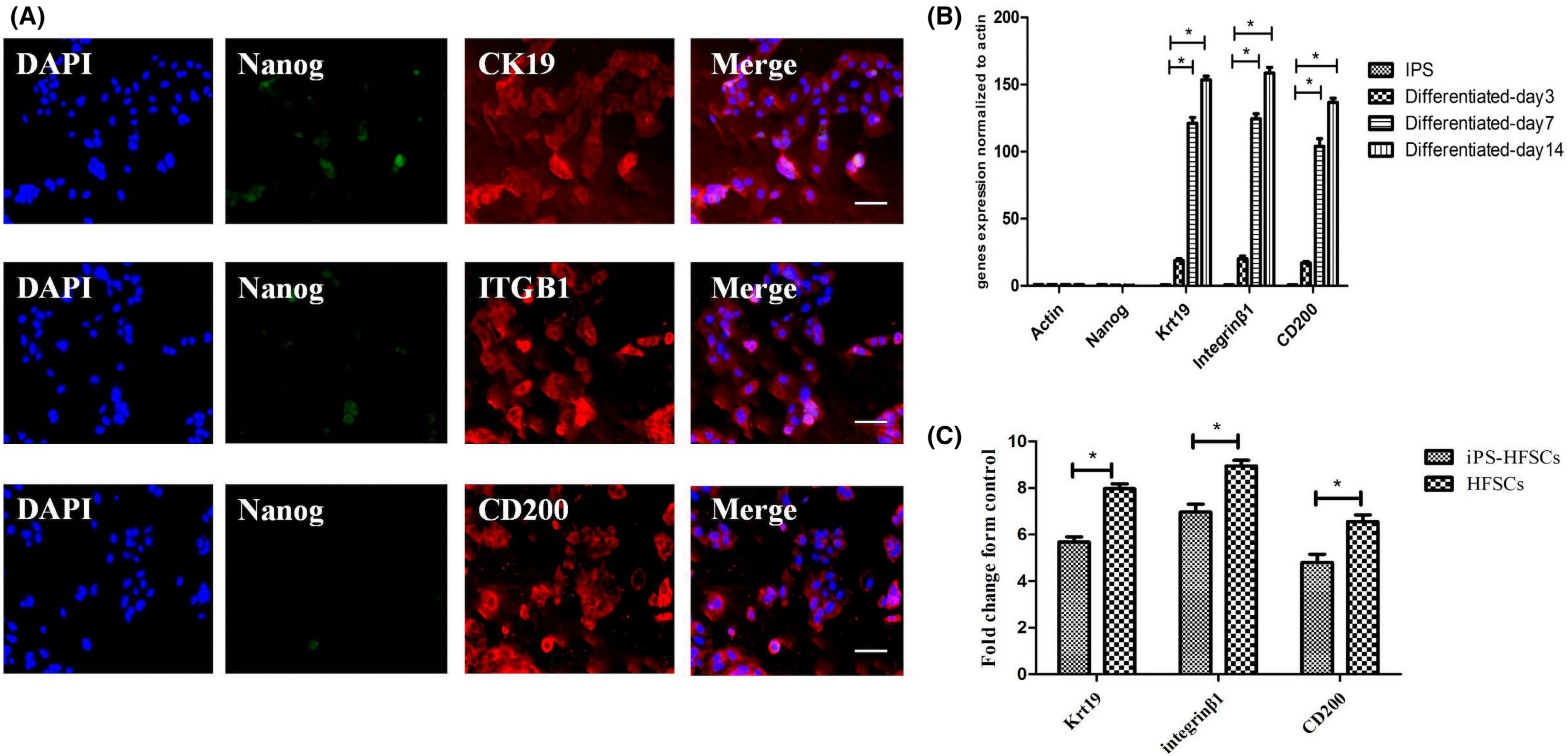
(A) Immunofluorescence staining of HFSCs specific markers. Scale bar, 50 μm. (B) Expression of relevant target genes after iPSCs induction at different time points. (C) Expression of relevant target genes after 14 days of iPSCs induction compared with human hair follicle stem cells.

HFSCs markers cytoplasmic protein CK19 was positively expressed (red fluorescence), cell membrane protein integrinβ1 and CD200 were positively expressed (red fluorescence), while the allosteric gene Nanog was not expressed in the induced iPSCs (faint green fluorescence). The identification of specific markers further demonstrated that we have obtained cells with HFSCs phenotypes (Figure 3A).

#### 3.2.4 Results of RT‐qPCR assay

The RT‐qPCR results showed that the expression of HFSCs‐specific related genes (CK19, integrinβ1 and CD200) started to be upregulated after 3 days of iPSC induction, while the expression of iPSC allosteric genes Nanog started to be downregulated. The above HFSCs‐specific related genes expressions were further up‐regulated at 7 and 14 days of induction, in a time‐dependent manner, with increasingly adequate induction as the induction time progressed (Figure 3B). And the cells induced at 14days were compared with the expression of human‐derived HFSCs specific genes (CK19, integrinβ1 and CD200), which were found to be very close to each other and completely different from iPSCs (Figure 3C). The above results further indicate that we have obtained cells with the phenotype of HFSCs.

### 3.3 Preparation of astragalus polysaccharide‐containing 3D printed scaffolds and co‐culture with VEGF_165_ gene modified iPS‐HFSCs^GFP^

#### 3.3.1 Astragalus polysaccharide on cell proliferation ability assay and analysis of optimal concentration

**FIGURE 4:**
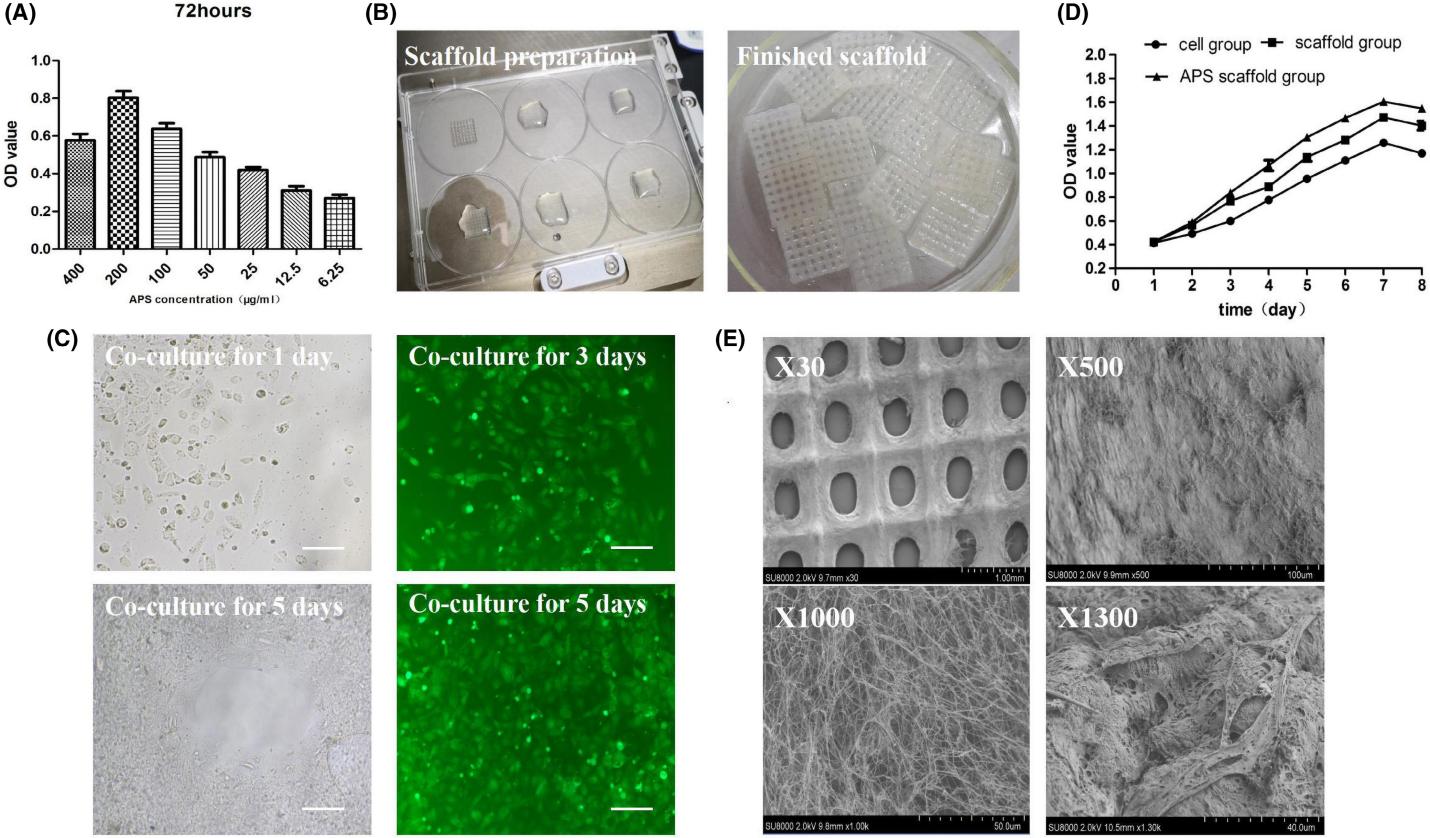
(A) Proliferation inhibition of VEGF_165_ gene‐modified iPS‐HFSCs^GFP^ by different concentrations of astragalus polysaccharide. (B) Naked eye view of astragalus polysaccharide‐collagen‐sodium alginate‐silk fibroin 3D printed degradable skin scaffolds. (C) Microscopic observation of astragalus polysaccharide‐containing 3D printed degradable scaffolds co‐cultured with VEGF_165_ gene‐modified iPS‐HFSCs^GFP^, scale bar, 100 μm. (D) Growth curves of different groups of VEGF_165_ gene‐modified iPS‐HFSCs^GFP^. (E) Scanning electron microscopy observation of astragalus polysaccharide‐containing 3D printed scaffold and co‐culture with VEGF_165_ gene‐modified iPS‐HFSCs^GFP^.

After 72 h culture effect, when the concentration of astragalus polysaccharide was between 25 and 400 μg/mL, the proliferation of VEGF_165_ gene modified iPS‐HFSCs^GFP^ were obvious, and the cell proliferation efficiency was positively correlated with the concentration of astragalus polysaccharide, especially the proliferation reached the peak at the concentration of 200 μg/mL, and the OD value was 0.80 ± 0.07. The above results suggest that our astragalus polysaccharide can effectively promote the proliferation of VEGF_165_ gene‐modified iPS‐HFSCs^GFP^, and 200 μg/mL astragalus polysaccharide is the relative optimal concentration, which can be prepared for the preparation of astragalus polysaccharide‐containing 3D printed degradable skin scaffolds. (Figure 4A).

#### 3.3.2 Preparation results of astragalus polysaccharide‐collagen‐sodium alginate‐silk fibroin 3D printed degradable skin scaffold

The whole scaffold was transparent, with good formability, consistent scaffold pore size, uniform spacing, consistent alignment, uniform thickness, and certain mechanical properties, which could withstand the extrusion and stretching of forceps without deformation and fracture (Figure 4B). It indicates that we successfully prepared good astragalus polysaccharide‐containing 3D printed degradable skin scaffolds.

#### 3.3.3 Results of co‐culture of astragalus polysaccharide‐containing 3D printed degradable skin scaffolds with cells

After the first day of culture, VEGF_165_ gene‐modified iPS‐HFSCs^GFP^ grew pavement‐like against the wall on the scaffold in a 6‐well plate, and the cells were in good condition. After the third day of culture, VEGF_165_ gene‐modified iPS‐HFSCs^GFP^ proliferated further on the scaffold and the green fluorescence of the cells did not diminish. After the fifth day of culture, the VEGF_165_ gene‐modified iPS‐HFSCs^GFP^ cells grew all over the scaffold, and the green fluorescence intensity of the cells was still highly expressed (Figure 4C). The above indicates that the astragalus polysaccharide‐containing 3D printed degradable skin scaffold is suitable for benign cell growth.

#### 3.3.4 Results of cell proliferation ability assay after scaffold cell co‐culture

The cell group, the empty scaffold group, and the scaffold group containing astragalus polysaccharide were relatively silent in the starting 2 days. On the third day, all three groups of cells began to proliferate significantly clonally and were in the logarithmic growth phase from day 4 to 7. On day seven, the OD value of cells in the astragalus polysaccharide‐containing scaffold group was the highest, reaching 1.61 ± 0.03. The proliferation ability of all three groups of cells started to decrease on the 8th day (Figure 4D). Therefore, the astragalus polysaccharide‐containing scaffold were selected after co‐culture for 5 days for in vivo experiment. The growth curves of all three groups of cells showed an “S” shape within 8 days, and the cell proliferation ability of the astragalus polysaccharide‐containing scaffold group was the strongest, suggesting that the 200 μg/mL astragalus polysaccharide‐containing scaffold was more favourable to the growth of VEGF_165_ gene‐modified iPS‐HFSCs^GFP^, indicating the good compatibility of the above scaffold and cells.

#### 3.3.5 Scanning electron microscopy detection of scaffold and cell ultrastructure results

Scanning electron microscopy showed uniform pores of the astragalus polysaccharide‐containing 3D printed scaffolds with uniform pore spacing, intact scaffolds and fine texture (magnification ×30, Figure 4E). Further local magnification observation showed that the scaffold was enriched with a honeycomb‐like mesh structure, which was porous and dense and interconnected, and the internal distribution was three‐dimensional, which was conducive to cell adhesion growth (magnification ×500 and ×1000, Figure 4E). Under high magnification, the VEGF_165_ gene‐modified iPS‐HFSCs^GFP^ were seen to adhere to the surface of the astragalus polysaccharide‐containing 3D printed scaffold material and grew in a polygonal spread with synapses between the cells, spanning the pores on the surface of the material, with good cellular status and excellent refractive index (magnification ×1300, Figure 4E). The above results indicate that the scaffolds have good biocompatibility.

### 3.4 VEGF_165_ gene‐modified iPS‐HFSCs^GFP^ compounded with astragalus polysaccharide‐containing 3D printed skin scaffolds for repairing whole skin defects in nude mice

#### 3.4.1 Nude mouse model construction and transplantation

**FIGURE 5:**
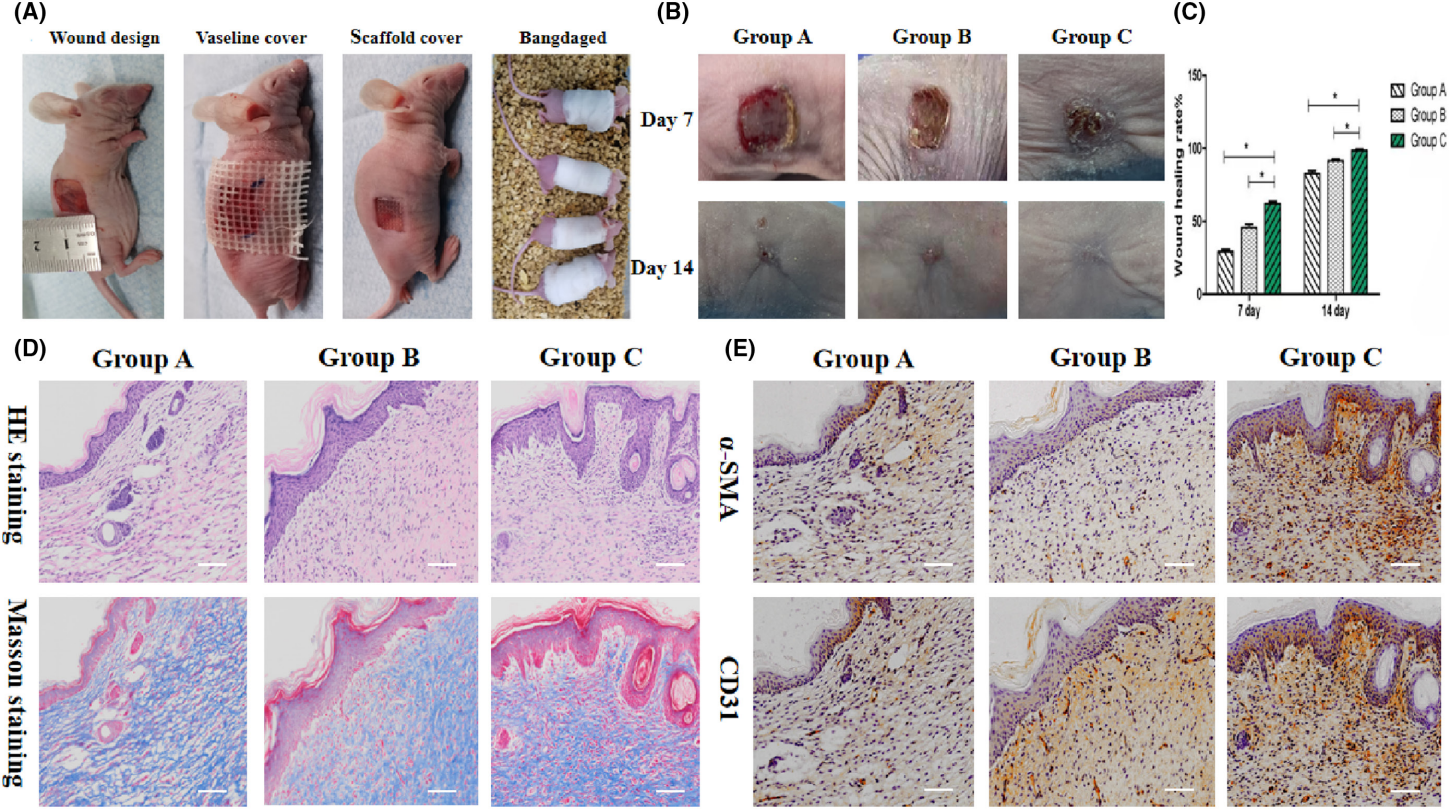
(A) Whole skin defect models and interventions in nude mice. (B) Visualization of wound healing after 7 and 14 days in the three different intervention models. (C) Comparison of wound healing rates after 7 and 14 days in the three groups with different intervention modes. (D) Histological examination of wounds after 14 days in the three groups with different intervention modalities. Scale bar, 100 μm. (E) Immunohistochemical detection of wounds after 14 days in the three groups with different intervention modes. Scale bar, 100 μm.

A 1.0 × 1.0 cm square full‐layer skin defect model was successfully prepared, and the blank control group A (covered with petroleum jelly), empty scaffold group B (3D‐printed skin scaffold containing astragalus polysaccharide) and tissue‐engineered skin group (VEGF_165_ gene‐modified iPS‐HFSCs^GFP^ + 3D printed skin scaffold containing astragalus polysaccharide) used skin scaffolds to cover the trauma, and all groups of nude mice were transplanted to complete Afterwards, the surgical area was wrapped with disposable sterile gauze and bandages (Figure 5A).

#### 3.4.2 Wound healing

The visualization of the trauma on the back of the nude mice showed that the trauma in the tissue‐engineered skin group shrank more significantly after 7 days, and the trauma was dry and crusted while new skin tissue was formed. 14 days later, the skin repair of the trauma in all groups improved significantly, and almost all of the trauma in the tissue‐engineered skin group was healed and new skin tissue was formed (Figure 5B). The wound healing rate of the tissue‐engineered skin group increased the fastest after 7 days. After 14 days, the wound healing rate of each group increased substantially and was close to complete healing, with the tissue‐engineered skin group > empty scaffold group > blank control group. These results indicated that the tissue‐engineered skin covering the wound further accelerated wound healing (Figure 5C).

#### 3.4.3 Histological test results

The tissue‐engineered skin group showed proliferation of epithelial cells and fibroblasts, dense arrangement of collagen fibres, abundant myofibers, presence of intact new hair follicles, and the moderate thickness of the new dermis, which was better than the other two groups. This indicates that tissue‐engineered skin can increase collagen formation on the traumatic surface, increase the thickness of the new dermis, and reconstruct skin hair follicles to improve the quality of regenerative repair of defective skin (Figure 5D).

#### 3.4.4 Immunohistochemical test results

The expression of α‐SMA and CD31 in the tissue‐engineered skin group was significantly better than that in the empty scaffold group and the blank control group in the new skin. It indicated that the tissue‐engineered skin group had higher intertissued collagen and vascular neovascularization than the other two groups and abundant vascular maturation (Figure 5E).

#### 3.4.5 RT‐qPCR related gene detection results

**FIGURE 6:**
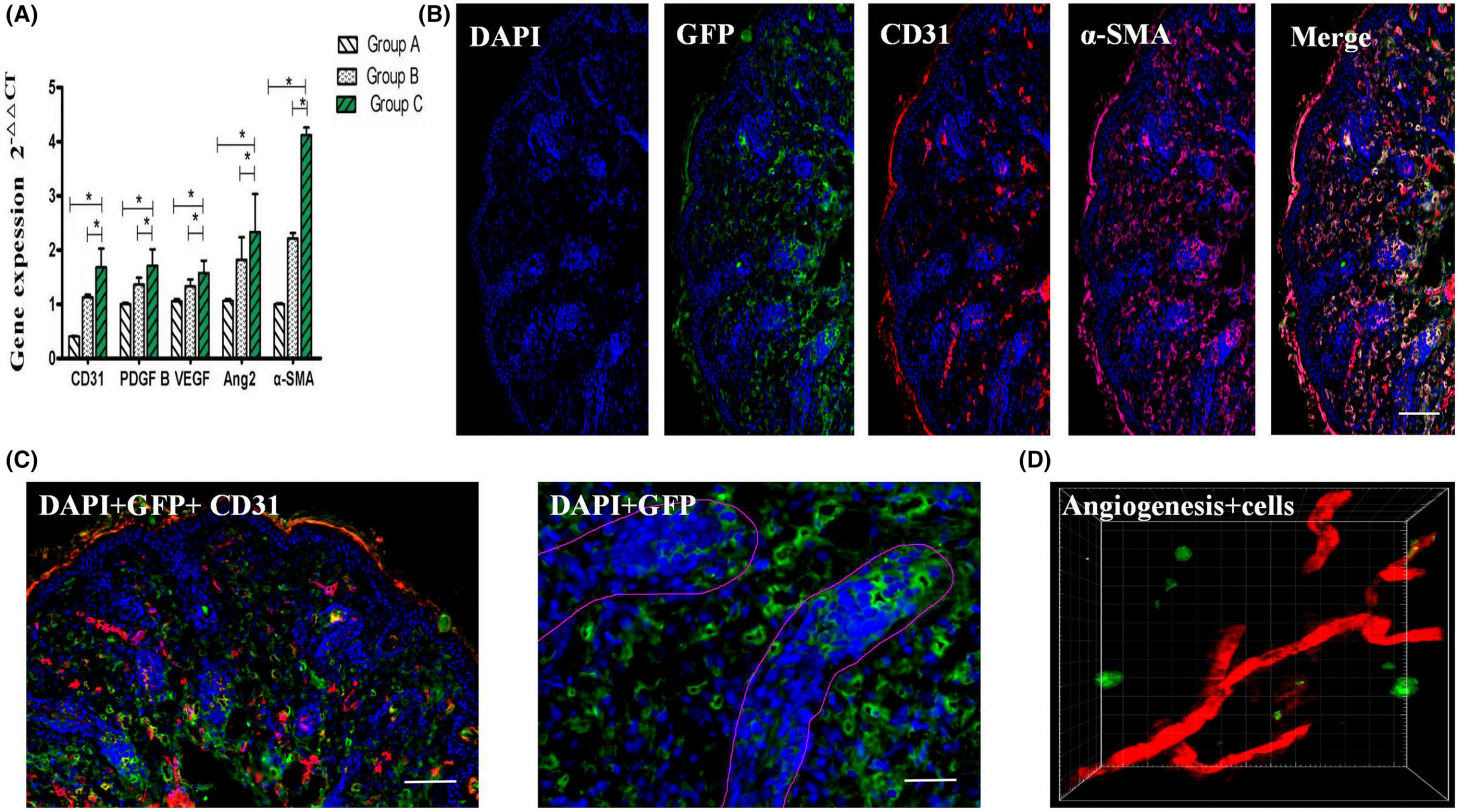
(A) Trauma‐related genes detected after 14 days in the three groups with different intervention modes. (B, C) Immunofluorescence detection of trauma in the tissue‐engineered skin group after 14 days. Scale bars, 200 μm and 100 μm. (D) Three‐dimensional reconstruction of neovascularization and VEGF_165_ gene‐modified iPS‐HFSCs^GFP^ in the healing skin area of living nude mice in the tissue‐engineered skin group.

The gene expressions of CD31, PDGF B, VEGF, Ang2 and α‐SMA in the tissue‐engineered skin group were significantly better than those in the empty scaffold group and the blank control group in the new skin (*p* < 0.05), and the α‐SMA gene expressions were most significantly increased. It further suggested that tissue‐engineered skin may be able to accelerate the remodelling of the nutritional internal environment of the injured skin by accelerating collagen and vascularization of the traumatized surface, and eventually achieve a more complete structural and functional new skin reconstruction (Figure 6A).

#### 3.4.6 Immunofluorescence assay results

A small number of VEGF_165_ gene‐modified iPS‐HFSCs^GFP^ (green fluorescence) in the tissue‐engineered skin group were located in the endothelium of the neovascularized official lumen and expressed the vascular endothelial cell marker CD31 (red fluorescence). More VEGF_165_ gene‐modified iPS‐HFSCs^GFP^ (green fluorescence) were consistent with the location of collagen marker α‐SMA (purple fluorescence) expression in the neonatal skin (Figure 6B). The above results indicated that most of the VEGF_165_ gene‐modified iPS‐HFSCs^GFP^ were directly involved in intertissued collagen neogenesis, and a few were directly involved in angiogenesis. The VEGF_165_ gene‐modified iPS‐HFSCs^GFP^ (green fluorescence) were mainly concentrated around the new hair follicles, and a small portion of them was distributed in the basal bulge of the new hair follicles, which indicated that the VEGF_165_ gene‐modified iPS‐HFSCs^GFP^ could also be directly involved in the formation of new hair follicles with bulges, and most of them might promote the reconstruction of the hair follicles in the host new skin (Figure 6C).

#### 3.4.7 Three‐dimensional reconstruction of neovascularization and VEGF_165_ gene‐modified iPS‐HFSCs^GFP^

The neovascularization of the healing skin area was clearly visible (red fluorescence), and the VEGF_165_ gene‐modified iPS‐HFSCs^GFP^ were mainly distributed around the neovascularization (green fluorescence), which visually verified the results of immunofluorescence detection and further confirmed the role of VEGF_165_ gene‐modified iPS‐HFSCs^GFP^ in skin healing to promote early vascularization of the trauma, and this accelerating effect was mainly achieved by accelerating host‐derived angiogenesis (Figure 6D).

## 4 DISCUSSION

The most studied stem cells, such as embryonic stem cells, bone marrow mesenchymal stem cells and adipose stem cells, have the potential for multidirectional differentiation and high proliferation capacity. However, they also have their disadvantages: ethical and moral restrictions, inconvenience in obtaining materials, injure donors, limited sources and numbers, and high immune rejection of allogeneic transplantation, which inevitably slow down the rapid development of regenerative medicine.^41^, ^42^, ^43^, ^44^ iPSCs hold great therapeutic promise for regenerative medicine. iPSCs can proliferate indefinitely in culture and differentiate into nearly all cell types. Thus this provides unlimited source material for cell‐based therapies to treat a wide range of diseases.

In terms of induced differentiation of extra‐embryonic cells into iPSCs, the sources can be divided into adult stem cells (bone marrow mesenchymal stem cells,^45^, ^46^ adipose mesenchymal stem cells^47^, ^48^ and hair follicle mesenchymal stem cells,^49^ etc.) and adult cells (peripheral blood cells,^50^ lymphocytes^51^, ^52^ and skin fibroblasts,^53^, ^54^ etc.). However, the reprogramming efficiency, induction factors, induction time of the induction medium and multi‐directional differentiation potential of adult cells can differ greatly depending on their tissue origin. Skin fibroblasts are easy to obtain and are an early source of adult seed cells that have been studied for transformation to iPSCs. Given the previous research base and mature induction system of our group, we used the classical OCT4, SOX2, C‐MYC and KLF4 four‐factor induction method. We also used a non‐integrated recombinant plasmid electrotransfer method to obtain a normal human skin fibroblast‐derived iPSCs line. Compared with traditional lentiviral vector and retroviral vector treatments, this approach is relatively simple to operate, which has high transfection efficiency, and reduces the chance of causing insertional mutations, thus reducing the risk of tumorigenesis and transplantation immune rejection.

In our group, we obtained hair follicle stem cells from mouse tentacles in a previous study to establish a culture system for the efficient acquisition of hair follicle stem cells.^38^, ^55^ However, as in our study, most hair follicle stem cells are currently obtained from various animal or need to be cultured by isolating the hair follicle structure of the human scalp and attaching the tissue block to the wall or digesting it, and some need to directly pluck the hair and hair follicle for separation and extraction, which can easily destroy the structure of the hair follicle bulge and the efficiency of culturing primary cells is low, and there are always inconvenience in obtaining the material, easy to injure the donor, long culture cycle, limited source and quantity, and allogeneic cells. This is often difficult to meet the demand for a large number of high‐purity hair follicle stem cells for tissue‐engineered skin, so scholars attempted to explore and study other ways for obtaining seed cells (hair follicle stem cells).^56^, ^57^ The possibility of induced differentiation of iPSCs into hair follicle stem cells is an important approach to solving the above problems. Hence, our group used the characteristic advantages of iPSCs to construct an iPSC‐derived hair follicle stem cell system.

The current literature reports the successful differentiation of iPSCs induced into hair follicle stem cells is mainly divided into suspension and adherent culture systems.^58^, ^59^ Our group's previous study of the suspension culture system found that iPSCs induced CD200^+^/ITGA6^+^ epithelial stem cells exhibited hair follicle stem cell phenotype, but the positive rate was still only about 20%, and the induction protocol was cumbersome and cell viability decreased significantly during the induction process. We modified the protocol of Zhou et al.^38^ by using basal culture A (KSFM + 0.2 ng/mL EGF + 30 μg/mL BPE + 1% penicillin–streptomycin + 10 ng/mL BMP‐4 + 1 μM RA) for the adnexal iPSCs induction, and then 0.25 μM valproic acid was added to culture medium A after 7 days. After 14 days of induction, iPS‐HFSCs were purified using the differential apposition method using type IV collagen‐coated culture dishes. After a relatively simple induction, the cells showed the typical pavement morphology of hair follicle stem cells with good stereoscopic sense and high refractive index and had more typical biological properties of hair follicle stem cells. A comparison of the expression of genes and proteins specific to human‐derived hair follicle stem cells (CK19, integrinβ1 and CD200) in the cells after 14 days of induction showed that both cells were very close and completely different from iPSCs. It showed that iPSCs after induction had taken on hair follicle stem cell‐like characteristics.

Astragalus polysaccharide, as the main active substance in astragalus, has an excellent performance in anti‐inflammatory, vascular protection and pro‐angiogenesis. Zhang et al.^60^ found that astragalus polysaccharide significantly enhanced the migration and tube formation of vascular endothelial cells through in vitro cellular and in vivo experiments with intrathecal injection of astragalus polysaccharide in sciatic nerve injury. Key proteins in the VEGF pathway were found by RNA‐seq AKT and eNOS were enriched with a large number of differential genes, suggesting that the role of astragalus polysaccharide in angiogenesis may be related to the activation of the AKT/eNOS signalling pathway. There are no reports related to the proliferative cloning ability of astragalus polysaccharides with hair follicle stem cells and the co‐culture of iPSC‐derived hair follicle stem cells with 3D printed skin containing astragalus polysaccharides. Our results showed that astragalus polysaccharide could effectively promote the proliferation of VEGF_165_ gene‐modified iPS‐HFSCs^GFP^, and 200 μg/mL astragalus polysaccharide was the relative optimal concentration. The scaffold containing 200 μg/mL astragalus polysaccharide was more favourable for the growth of VEGF_165_ gene‐modified iPS‐HFSCs^GFP^. Scanning electron microscopy showed that the ultrastructure of the astragalus polysaccharide‐containing 3D printed scaffold was excellent and suitable for the healthy proliferation of VEGF_165_ gene‐modified iPS‐HFSCs^GFP^, and both were biocompatible.

To verify the effect of iPS‐HFSCs compounded with astragalus polysaccharide‐containing 3D printed degradable scaffold on the regenerative repair of total skin defects, to elucidate its form of action in promoting vascular neovascularization in nude mice, and to explore its mechanism of action in promoting regenerative repair of total skin defects, we designed the VEGF_165_ gene‐modified iPS‐HFSCs^GFP^ compounded with 200 μg/mL astragalus polysaccharide‐containing 3D printed degradable scaffold. Polysaccharide 3D printed degradable scaffolds containing 200 μg/mL astragalus were implanted into the dorsal whole skin defect area of nude mice for the study. Histopathologically, the tissue‐engineered skin in this study was found to increase trabecular collagen formation, increase the thickness of the new dermis, and reconstruct skin follicles, improving the quality of regenerative repair of the defective skin. The results of RT‐qPCR and Western blot assay of neonatal skin tissue samples also suggested that VEGF_165_ gene‐modified iPS‐HFSCs^GFP^ were most directly involved in intertissued collagen neogenesis, and a small portion was directly involved in angiogenesis, which is consistent with the results of 3D reconstruction assay of neovascular and VEGF_165_ gene‐modified iPS‐HFSCs^GFP^. We also found that VEGF_165_ gene‐modified iPS‐HFSCs^GFP^ were mainly distributed around the new hair follicles and a small portion was distributed in the basal bulge of the new hair follicles, suggesting that VEGF_165_ gene‐modified iPS‐HFSCs^GFP^ could also directly participate in the formation of new hair follicles with bulges, thus promoting the reconstruction of new hair follicles in the host skin.

## 5 CONCLUSION

**FIGURE 7:**
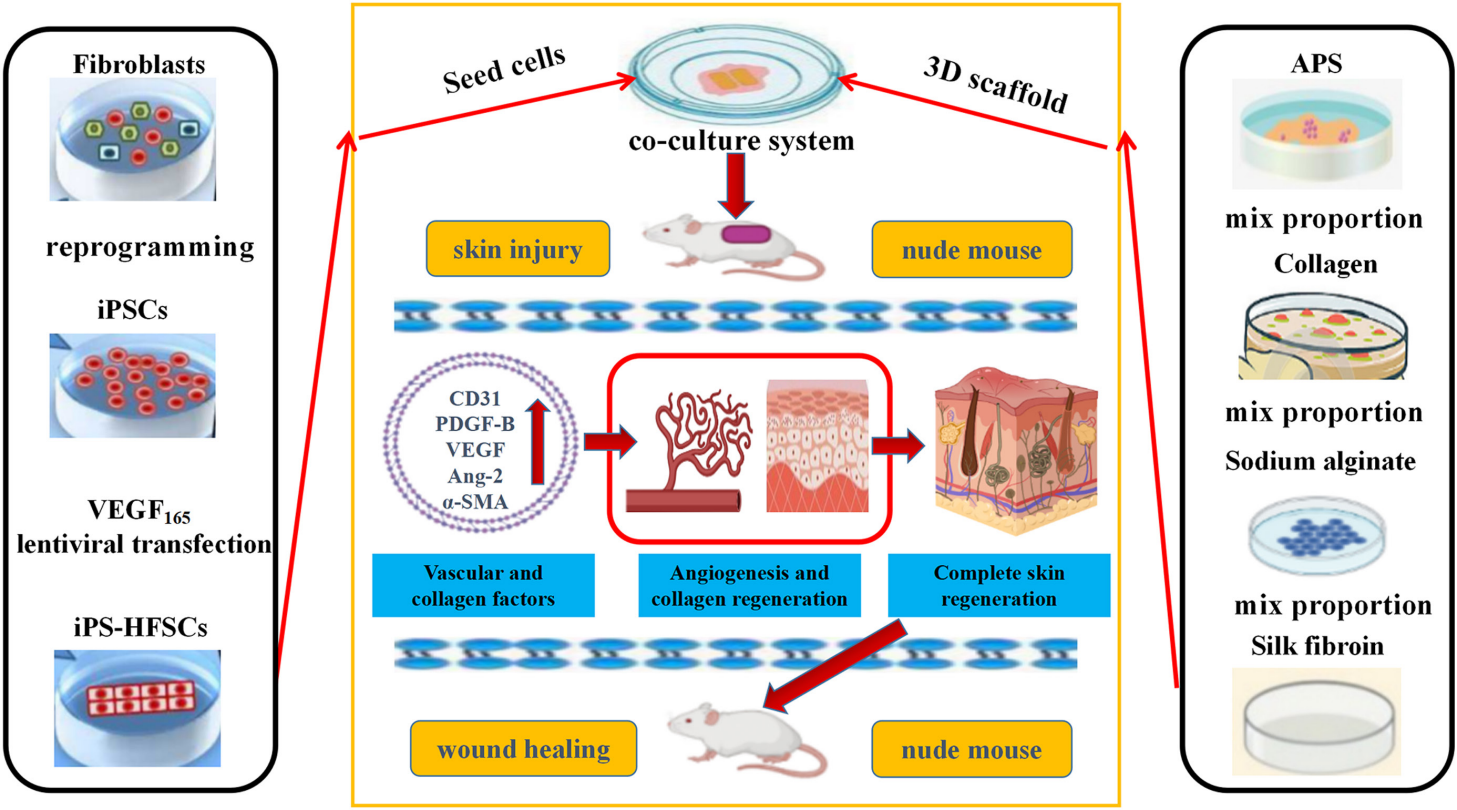
Schematic diagram of repairing full‐thickness skin defects by CK19^+^/Integrinβ1^+^/CD200^+^ VEGF_165_ gene modified iPS‐HFSCs^GFP^ seeded on a 3D printed degradable scaffold containing 200 μg/mL astragalus polysaccharide.

In summary, as shown in Figure 7, VEGF_165_ gene‐modified iPS‐HFSCs^GFP^ composite containing 200 μg/mL astragalus polysaccharide 3D printed degradable scaffold implanted in the area of total dorsal skin defect in nude mice is through activation of key factors closely related to host trabecular vascularity (CD31, PDGF B, VEGF, Ang2) and collagen fibril key factor α‐SMA, and with different amounts of cells directly involved in the neovascularization of blood vessels, collagen and hair follicles in the new skin, accelerating the reconstruction of the trabecular nutrient blood supply environment, accelerating the synthesis of collagen, remodelling regional hair, and achieving a more complete structural and functional skin regenerative repair. This provides a new idea for the application of tissue‐engineered skin in the repair of clinical skin defects treatment.

## AUTHOR CONTRIBUTIONS

**Weibin Du:** Formal analysis (lead); funding acquisition (lead); project administration (lead); writing – original draft (lead). **Jintao Hu:** Data curation (equal); methodology (equal); validation (equal); writing – original draft (equal). **Zhenwei Wang:** Conceptualization (equal); formal analysis (equal); writing – original draft (equal). **Xiaolong Huang:** Resources (supporting); validation (supporting). **Huateng Zhou:** Data curation (supporting); formal analysis (supporting); resources (supporting). **Yadong Yang:** Conceptualization (supporting); methodology (supporting); software (supporting). **Huahui Hu:** Formal analysis (supporting); validation (supporting); visualization (supporting). **Rongliang Chen:** Conceptualization (supporting); data curation (supporting); formal analysis (supporting). **Fuxiang Shen:** Supervision (supporting); writing – review and editing (supporting). **Renfu Quan:** Investigation (equal); project administration (equal); supervision (equal); writing – review and editing (equal).

## FUNDING INFORMATION

This work is supported by National Natural Science Foundation of China (No. 81904053). Special Research Project of the Affiliated Hospital of Zhejiang Chinese Medical University (No. 2021FSYYZY43). Hangzhou Medical and Health Technology Planning Project (Nos. B20220021, B20200032, A20220507), Hangzhou Science and Technology Planning Project (Nos. 2020ZDSJ0042, 20220919Y084). Zhejiang Province Traditional Chinese Medicine Science and Technology Project (Nos. 2022ZB232, 2023ZR046), Research Project of Zhejiang Chinese Medical University (No. 2021JKZKTS057B), Hangzhou Xiaoshan District Science and Technology Planning project (No. 2019216). The funding body played no role in the design of the study and collection, analysis, and interpretation of data and in writing the manuscript.

## CONFLICT OF INTEREST STATEMENT

All the authors declare that they have no conflicts of interest.

## Data Availability

All data generated and/or analysed during this study are included in this published article.
